# Evaluation of the Broad-Range PCR/ESI-MS Technology in Blood Specimens for the Molecular Diagnosis of Bloodstream Infections

**DOI:** 10.1371/journal.pone.0140865

**Published:** 2015-10-16

**Authors:** Elena Jordana-Lluch, Montserrat Giménez, Mª Dolores Quesada, Belén Rivaya, Clara Marcó, Mª Jesús Domínguez, Fernando Arméstar, Elisa Martró, Vicente Ausina

**Affiliations:** 1 Microbiology Service, Germans Trias i Pujol University Hospital, Department of Genetics and Microbiology, Autonomous University of Barcelona, Badalona, Spain; 2 CIBER in Respiratory Diseases (CIBERES), Madrid, Spain; 3 Emergency Room, Germans Trias i Pujol University Hospital, Badalona, Spain; 4 Intensive Care Unit, Germans Trias i Pujol University Hospital, Badalona, Spain; 5 Health Sciences Research Institute (IGTP), Badalona, Spain; 6 CIBER in Epidemiology and Public Health (CIBERESP), Madrid, Spain; The Foundation for Medical Research, INDIA

## Abstract

**Background:**

Rapid identification of the etiological agent in bloodstream infections is of vital importance for the early administration of the most appropriate antibiotic therapy. Molecular methods may offer an advantage to current culture-based microbiological diagnosis. The goal of this study was to evaluate the performance of IRIDICA, a platform based on universal genetic amplification followed by mass spectrometry (PCR/ESI-MS) for the molecular diagnosis of sepsis-related pathogens directly from the patient’s blood.

**Methods:**

A total of 410 whole blood specimens from patients admitted to Emergency Room (ER) and Intensive Care Unit (ICU) with clinical suspicion of sepsis were tested with the IRIDICA BAC BSI Assay (broad identification of bacteria and *Candida* spp.). Microorganisms grown in culture and detected by IRIDICA were compared considering blood culture as gold standard. When discrepancies were found, clinical records and results from other cultures were taken into consideration (clinical infection criterion).

**Results:**

The overall positive and negative agreement of IRIDICA with blood culture in the analysis by specimen was 74.8% and 78.6%, respectively, rising to 76.9% and 87.2% respectively, when compared with the clinical infection criterion. Interestingly, IRIDICA detected 41 clinically significant microorganisms missed by culture, most of them from patients under antimicrobial treatment. Of special interest were the detections of one *Mycoplasma hominis* and two *Mycobacterium simiae* in immunocompromised patients. When ICU patients were analyzed separately, sensitivity, specificity, positive and negative predictive values compared with blood culture were 83.3%, 78.6%, 33.9% and 97.3% respectively, and 90.5%, 87.2%, 64.4% and 97.3% respectively, in comparison with the clinical infection criterion.

**Conclusions:**

IRIDICA is a promising technology that offers an early and reliable identification of a wide variety of pathogens directly from the patient’s blood within 6h, which brings the opportunity to improve management of septic patients, especially for those critically ill admitted to the ICU.

## Introduction

Bloodstream infection is a life-threatening illness due to the presence of microorganisms or their toxins in the blood [1]. The systemic deleterious host response to this infection can lead to severe sepsis and septic shock, which affect millions of people around the world with an increasing incidence [2]. Once the symptoms are recognized, the administration of antibiotic therapy during the first hour is strongly recommended [2], as every hour gained in initiating proper antimicrobial therapy significantly increases the probability of patient survival [3]. However, the current gold standard still relies on culture, which may take up to 3 days before obtaining the identification and performing the antimicrobial susceptibility testing. Thus, rapid identification of the causal agent directly from the patient’s blood would be desirable, as it would allow clinicians to readdress the initial antibiotic therapy if necessary, before culture-based identification and susceptibility testing results are available.

A few years ago, an innovative technology based on universal PCR amplification coupled with mass spectrometry was described (PCR/ESI-MS) [4,5]. The first version of this technology, although promising, showed a moderate sensitivity ranging from 50% to 68% for the diagnosis of bloodstream infections (in comparison with blood culture results plus other microbiological findings) [6,7]. A newer version of this technology called IRIDICA (Ibis Biosciences, Carlsbad, CA) is in development. Its main improvement is an enhanced sensitivity, up to 83–91%, due to an increase in the volume of blood tested (5 mL instead of 1.25 mL in the former version), the optimization of PCR conditions and reagents to be tolerant of high loads of human DNA, and an improved downstream processing and analysis step to ensure high sensitivity [8]. The goal of this study was to analyze the clinical performance of this new platform for the diagnosis of bloodstream infections as well as its ability for identifying a wide range of pathogens.

## Materials and Methods

### Ethics statement

Written informed consent was obtained from all patients or their guardians. This study was approved by the Clinical Research Ethics Committee at Germans Trias i Pujol University Hospital (“Comité Ético de Investigación Clínica”, CEIC).

### Patients and specimens

**Fig 1:**
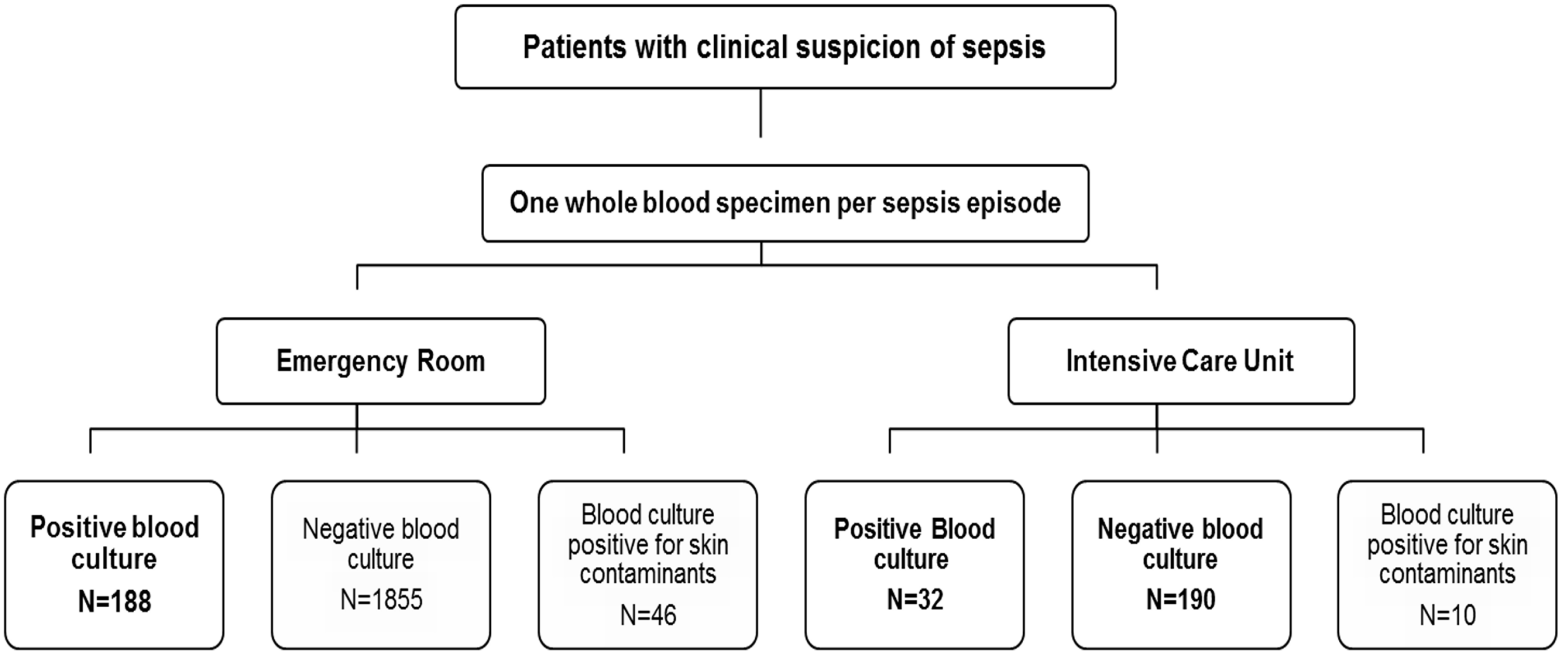
Flowchart depicting the study design.

This was an observational prospective study including a total of 405 patients admitted to the ICU or ER (median age 66 years, range 16–101; 246 male and 161 female) with a suspicion of sepsis according to the American College of Chest Physicians/Society of Critical Care Medicine (ACCP/SCCM) criteria [9], and enrolled between September 2012 and March 2013 at a tertiary care center in Spain (Fig 1). For each patient, one extra whole blood specimen was collected in an EDTA tube under aseptic conditions at the same time as the inoculation of the blood culture for routine microbiological testing (at the onset of fever or other clinical signs of sepsis). For five patients, a blood specimen from two different sepsis episodes was included adding up to 410 specimens. The result of the paired blood culture for each specimen was recorded. Over the study period, a blood specimen was obtained for 222 ICU patients (median age 57.7 years, range 16–83; 138 male, 82 female), which were all tested by IRIDICA. These corresponded to 32 specimens with a paired positive blood culture and 190 with a paired negative blood culture. In order to further assess the ability of the technology for identifying a wider range of pathogens, we also included 188 specimens with paired positive blood culture from ER patients (median 71.5 years, range 20–101; 110 male, 80 female), as this clinical unit is the source of 60% of blood cultures sent to the Microbiology laboratory. Those patients in which skin contaminants were identified by blood culture were excluded from this study (*n* = 56 cases with 59 microorganisms isolated from a single positive culture bottle with coagulase-negative staphylococci (*n* = 46), *Streptococcus* spp. (*n* = 5), *Micrococcus* spp. (*n* = 3), *Corynebacterium* spp. (*n* = 2), *Bacillus* spp. (*n* = 1), *Propionibacterium* spp. (*n* = 1), and *Stenotrophomonas maltophilia* (*n* = 1)). Whole blood specimens were stored at -20°C until testing at Ibis Biosciences.

### Conventional microbiological methods

For each adult patient, a set of two blood cultures, including two aerobic and one anaerobic blood culture bottles, were inoculated with up to 10 mL of blood each. The blood culture bottles were incubated in the Bactec 9240 blood culture system (Becton Dickinson, Franklin Lakes, NJ, USA) for up to 5 days. The identification and susceptibility testing of the microorganisms were achieved using the Vitek-2 Compact system (BioMérieux, Marseille-L’Étoile, France) directly from positive blood culture bottles after performing a Gram stain and a concentration procedure [10,11]. Conventional cultures were also performed, following standard microbiological methods for identification and antibiotic susceptibility testing (disc diffusion and minimum inhibitory concentration methods) as required.

### Specimen processing with IRIDICA

Specimen testing with IRIDICA (Ibis Biosciences) was performed according to the manufacturer’s instructions using the IRIDICA BAC BSI Assay (Ibis Biosciences-Abbott Molecular (Des Plaines, IL). The work presented here was done using IRIDICA system under development. IRIDICA (CE-IVD) is now commercially available (http://iridica.abbott.com/). As previously described [8], this process includes automated DNA extraction, PCR set-up, PCR amplification, amplicon purification, and electrospray ionization time-of-flight mass spectrometry (PCR/ESI-MS), leading to microbial identification from whole blood in 6h. Computational matching of observed amplicon base compositions to a signature database provides broad-spectrum microbial identification. Briefly, 5 mL whole blood samples were chemically and mechanically lysed and an extraction control was added to each specimen for process monitoring purposes. DNA extraction and PCR set-up was automatically performed by a single instrument using pre-filled individual disposable sample preparation cartridges and pre-filled 16-well PCR reaction strips. The BAC BSI Assay utilizes several conserved-site primer pairs designed to amplify variable (and thereby discriminable) products from a broad range of bacteria and *Candida* spp., as well as primer pairs targeted to common antibiotic resistance loci conferring resistance to methicillin (*mecA*), vancomycin (*van*A and *van*B) and carbapenems (*KPC*). PCR products were then desalted and concentrated relative to human genomic DNA in an automated system and analyzed through ESI-MS. The base compositions of detected amplicon strands were deduced from the measured masses and compared with a reference database, leading to the identification of the microorganisms present in clinical samples. Internal calibrants present in each reaction allowed for relative (qualitative) approximation of target concentrations (expressed as levels), which in turn were used to limit noise- and contamination-derived background detections through thresholding of positive signals.

### Data interpretation and statistical analysis

For each specimen, the results obtained with IRIDICA were compared with those obtained using conventional methods (blood culture was considered the gold standard). When discrepancies between these methods were found, the clinical significance of the discrepant results was determined by comparison with a constructed “clinical infection criterion”; for this purpose, a clinical microbiologist together with a clinician were asked to retrospectively evaluate the discrepant results obtained by IRIDICA and to interpret them in the same way as the blood culture results are evaluated: the clinical records of the patients were reviewed in order to identify the diagnosed focus of infection, as well as the results of cultures from other specimens (i.e. microorganisms detected only by IRIDICA were considered true positives when the same microorganism had been isolated from a culture from another specimen type reflecting the focus of infection or supported by the nature of the underlying infection).

Since polymicrobial detections are not uncommon in bloodstream infections, the results obtained by IRIDICA and blood culture were compared at two levels using the two aforementioned gold standards: 1) by microorganism: a direct comparison for each microorganism isolated by conventional methods *vs*. the same microorganism detected by the molecular method, taking into consideration all microorganisms identified; and 2) by specimen: for each specimen with a single detection, matched positive or negative results by each method were recorded. In the latter case, specimens with polymicrobial detections were excluded, as they could not be properly classified (i.e. both methods agreed in some but not all microorganisms identified). In those terms, the Cohen’s Kappa coefficient of agreement by microorganism, and the positive and negative agreement by microorganism and by specimen were calculated with OpenEpi software [12]. In the ICU setting positive and negative agreements were equivalent to sensitivity and specificity, as all blood specimens consecutively obtained during the study period were tested (including specimens with both positive and negative paired blood culture). The positive and negative predictive values were also calculated both by microorganism and by specimen in this subgroup of patients. IRIDICA performance was compared between ICU and ER subgroups using the Pearson's chi-squared test (χ^2^). Clinical and molecular quantitative variables of interest were compared between groups using the Mann-Whitney U test (non-Normal distribution), and data was expressed as median and range. *P*-values <0.05 were considered significant. Statistical analyses were performed using the statistical software package SPSS v15.0.

## Results

Data analysis was performed on 408 specimens (220 from ICU and 188 from ER), as an invalid IRIDICA result was obtained in two cases due to the lack of detection of the extraction control.

### Overall agreement between both methods by microorganism

In comparison with blood culture, IRIDICA showed 73.3% positive concordance (detection of the same microorganism by the two methods) and 64.1% negative concordance (negative by the two methods) (Table 1). IRIDICA detected 80 microorganisms that did not grow in blood culture; 41 (51.2%) of these were supported by clinical facts (S1 Table). On the contrary, the presence of 7 (8.8%) microorganisms could not be supported by clinical evidence (S1 Table), and another 32 (40%) microorganisms were considered clinically irrelevant contaminants from the skin flora (i.e. coagulase-negative staphylococci, *Propionibacterium acnes*, etc.) or from the environment (i.e. *Methylobacterium* spp., *Pseudomonas putida*, etc.) (S2 Table). When the results were reanalyzed taking clinical information into consideration, the positive and negative agreement were, respectively, 77.2% and 78.6% (Table 1). All microorganisms with clinical significance were identified by IRIDICA at species level except for five (one *Acinetobacter baumannii* identified as *Acinetobacter* spp., one each *Streptococcus pneumoniae* and *Streptococcus viridans* group as *Streptococcus* spp., and two potential *Aspergillus* spp. as “Fungus detected, no identification can be provided”). The microorganisms with clinical significance isolated by culture or detected by IRIDICA are listed in Table 2.

**Table 1:** Agreement between methods according to the two gold standards used by microorganisms isolated by conventional microbiological methods and detected by IRIDICA.

	Global		Emergency Room		Intensive Care Unit	
	BC gold standard	Clinical infection criterion	BC gold standard	Clinical infection criterion	BC gold standard	Clinical infection criterion
**Matched positives (*n*)**	176	217^a^	147	152^a^	29	65 ^a^
**Matched negatives (*n*)**	143	143	n.a.	n.a.	143	143
**IRIDICA overcalls (*n*)**	80	39	21	16	59	23
**IRIDICA misses (*n*)**	64	64	56	56	8	8
**Overall agreement (%)**	68.9	77.8	n.a.	n.a.	72.0	87.0
**Positive agreement (%)**	73.3	77.2	72.4	73.1	78.4 ^b^	89.0 ^b^
**Negative agreement (%)**	64.1	78.6	n.a.	n.a.	70.8 ^b^	86.1 ^b^

^a^ IRIDICA overcalls with clinical significance were classified as matched positives according the clinical infection criterion.

^b^ Positive and negative agreement correspond to sensitivity and specificity, as all blood specimens consecutively obtained during the study period were tested (including specimens with both positive and negative paired blood culture).

n.a., not applicable.

**Table 2:** Microorganisms with clinical significance identified by either or both methods.

		Blood culture and IRIDICA	Blood culture only	IRIDICA only
**Gram-positive bacteria**	*Staphylococcus aureus*	8	3	3
	Coagulase-negative staphylococci	4	4	0
	*Streptococcus pneumoniae*	8	5	3
	Viridans streptococci	4	3	0
	*Granulicatella* spp.	1	0	0
	β-hemolytic *Streptococcus*	3	2	2
	*Enterococcus* spp.	15	6	1
	*Streptococcus gallolyticus*	1	*3*	1
	*Listeria monocytogenes*	1	2	0
	*Clostridium* spp.	0	1	0
	*Bacillus* spp.	0	1	0
	*Lactobacillus* spp.	0	1	0
	*Mycobacterium simiae*	0	0	2
	Subtotal	45	31	12
**Gram-negative bacteria**	*Escherichia coli*	85	18	7
	*Klebsiella pneumoniae/oxytoca*	13	0	5
	*Enterobacter cloacae/aerogenes*	10	3	3
	*Proteus mirabilis*	4	2	0
	*Salmonella enterica* sv. Enteritidis	1	0	0
	*Serratia marcescens*	1	1	0
	*Pseudomonas aeruginosa*	8	2	3
	*Pseudomonas* spp.	3	0	0
	*Stenotrophomonas maltophilia*	2	0	0
	*Acinetobacter baumannii*	1	0	0
	*Elisabethkingia meningoseptica*	1	0	0
	*Haemophilus influenzae*	0	1	1
	*Mycoplasma hominis*	0	0	1
	Subtotal	129	27	20
**Anaerobic bacteria**		0	1	4
**Fungi**	*Candida albicans*	0	1	2
	*Candida tropicalis*	1	0	1
	*Candida parapsilosis*	1	1	0
	*Candida glabrata*	0	1	0
	*Candida lusitanie*	0	1	0
	*Candida famata*	0	1	0
	Fungus detected, no ID provided	0	0	2
	Subtotal	2	5	5
**TOTAL**		**176**	**64**	**41**

### Overall agreement between both methods by specimen

Polymicrobial infections with clinical significance were detected by either or both methods in 28 out of 245 specimens (11.4%) (S3 Table). Given that both methods agreed in some but not all the microorganisms identified, these apparent polymicrobial samples were excluded from this analysis. Among the rest of specimens (*n* = 380), when blood culture was taken as the gold standard the positive and negative agreement of IRIDICA were 74.8% and 78.6% respectively, and those values rose to 76.9% and 87.2% when re-analyzed with the clinical infection criterion (Table 3).

**Table 3:** Agreement between methods according to the two gold standards used on specimens with a unique isolation/detection.

	Global		Emergency Room		Intensive Care Unit	
	BC gold standard	Clinical infection criterion	BC gold standard	Clinical infection criterion	BC gold standard	Clinical infection criterion
**Matched positives (*n*)**	148	166^a^	128	128	20	38 ^a^
**Matched negatives (*n*)**	143	143	n.a.	n.a.	143	143
**IRIDICA overcalls (*n*)**	39	21	0	0	39	21
**IRIDICA misses (*n*)**	50	50	46	46	4	4
**Overall agreement (%)**	76.6	81.3	n.a.	n.a.	79.1	87.9
**Positive agreement (%)**	74.8	76.9	73.6	73.6	83.3^b^	90.5 ^b^
**Negative agreement (%)**	78.6	87.2	n.a.	n.a.	78.6 ^b^	87.2 ^b^

^a^ IRIDICA overcalls with clinical significance were classified as matched positives according the Clinical infection Criterion.

^b^ Positive and negative agreement correspond to sensitivity and specificity, as all blood specimens consecutively obtained during the study period were tested (including specimens with both positive and negative paired blood culture).

n.a., not applicable.

### IRIDICA performance in ICU patients

In the analysis by microorganism, the overall agreement between methods was 72.0% (κ = 0.315) and the sensitivity, specificity and positive and negative predictive values of IRIDICA in comparison with blood culture were 78.4%, 70.8%, 33% and 95%, respectively (Table 1). When discrepancies found were evaluated using the clinical infection criterion (S1 Table), the overall agreement was 87% (κ = 0.711) and the values of analytical performance rose to 89%, 86.1%, 73.9% and 95%, respectively. Fourteen polymicrobial infections by either or both methods were excluded in order to perform the analysis by specimen (S3 Table). In those terms, the sensitivity, specificity and positive and negative predictive values in comparison with blood culture were 83.3%, 78.6%, 33.9% and 97.3% respectively, and rose to 90.5%, 87.2%, 64.4% and 97.3% respectively when considering the clinical infection criterion (Table 3).

### IRIDICA performance in ER patients

For this subgroup, we only included patients with a positive blood culture. According to the blood culture positivity rate in our center (8.2–12.2%), about 1860 patients with a negative culture would have had to be included in order to reach the 188 positive blood cultures tested, which was not feasible. From the 203 microorganisms isolated by culture, 147 were correctly detected by IRIDICA. Thus, the positive agreement by microorganism in comparison with blood culture was 72.4% (Table 1). A total of five microorganisms with clinical significance were detected by IRIDICA only, giving a positive agreement of 73.1% when the clinical infection criterion was used. When analyzed by specimen, the positive agreement was 73.6% either comparing with blood culture or clinical infection criterion (128 matched detections out of 174 monomicrobial infections) (Table 3).

### Assessment of factors potentially influencing performance of the molecular method

In those sepsis cases with a positive blood culture, several variables regarding both the culture (time to positivity) and the paired whole blood specimen (leukocyte count, storage time at -20°C) were compared between IRIDICA-negative and -positive specimens in order to find out if they were related to positivity by the molecular method. No statistically significant associations were found in the median white cell count between IRIDICA-negative and -positive specimens (11.9×10^6^ *vs*. 11.6×10^6^ cells/mL). Whole blood specimens had been stored for variable periods of time (range, 5–23 months; median, 13 months), but the median storage time was comparable between the two groups of specimens (12 months in IRIDICA negative *vs*. 13 months in IRIDICA positive). The difference in the time to positivity of the blood culture was marginally significant; specimens with an IRIDICA-positive result, tended to have a paired blood culture that was called positive earlier (the time to positivity of the blood culture was shorter: 14.5 *vs*. 15.5 h, *p* = 0.87). When comparing ICU *vs*. ER patients, the median of genomes per well by IRIDICA was significantly higher (*p* = 0.011) in the ICU group (median, 97 genomes/well; range, 15–229) than in the ER group (median, 24.5 genomes/well; range, 3–370).

### Detection of antibiotic resistance genes

The resistance markers detected by the molecular method in this study are depicted in Table 4. The resistance marker *mec*A was detected by IRIDICA in eight whole blood specimens, being four of them concordant with the result obtained with the conventional methods. From the other four detections, only one (*Staphylococcus aureus*, *mec*A) was supported by other microbiological findings, as the patient also had a bronchial aspirate positive for methicillin-resistant *S*. *aureus*. While none of the enterococci isolated and identified by conventional methods in this study were resistant to vancomycin or teicoplanin, IRIDICA detected the resistance markers *van*A and *van*B in two different specimens with an *Enterococcus faecium*.

**Table 4:** Description of the antibiotic resistance markers detected in this study.

Antibiotic	Identification and AST by conventional methods	Identification and resistance markers by IRIDICA
Methicillin	Coagulase-negative staphylococci, resistant	*Staphylococcus haemolyticus*, *Stahphylococcus epidermidis*, *mec*A
	*S*. *epidermidis*, resistant	*S*. *epidermidis*, *mec*A
	*Staphylococcus aureus* resistant	*S*. *aureus*, *mec*A
	Negative	*S*. *aureus*, *mec*A (clinically significant)
	Negative	*S*. *epidermidis*, *mec*A (contaminant)
	Negative	*S*. *hominis*, *mec*A (contaminant)
	Negative	*S*. *aureus*, *mec*A (no clinical explanation was found for this detection)
Vancomycin/ Teicoplanin	*Enterococcus faecium*, susceptible	*E*. *faecium*, *van*A
	*E*. *faecium*, susceptible	*E*. *faecium*, *van*B
Carbapenems	-	-

AST, antibiotic susceptibility testing.

## Discussion

Sepsis is a severe syndrome where time is crucial to the optimal patient management. There is a clear need to administer the appropriate antimicrobial therapy as soon as possible, as it will have a positive impact on patient’s survival [2,3]. However, the identification of the etiological agent from positive blood cultures may take 24–48h. Being able to detect and identify the causal pathogen directly from blood would speed the diagnosis and, therefore, improve the management of septic patients. Although several molecular methods designed for this purpose have been commercially available for several years, reported sensitivities are moderate in most cases and are not consistent across studies [13–17]. Given that in bloodstream infections microorganisms are present at low levels, working directly from small volumes of whole blood is an inherent limitation of molecular methods [17,18]. In order to overcome this issue, the new version of the PCR/ESI-MS technology, named IRIDICA, features improvements in the methodology and instruments, such as an increase in the volume of blood analyzed (5 mL), and the enrichment of microbial DNA during the purification process [8].

The overall agreement between IRIDICA and conventional methods was 68.9% when analyzing by microorganism and 76.6% when analyzing by specimen. Overall, 64 microorganisms isolated by conventional methods were not detected by IRIDICA (8 from ICU patients and 56 from ER patients). It should be borne in mind that molecular diagnostic assays typically use smaller volumes of blood (5 mL in this case) than blood culture (up to 30 mL), as the high amounts of human DNA found in whole blood (mostly in white blood cells) may hamper the detection of pathogen DNA. This fact could lead to the suboptimal sensitivities commonly reported for molecular methods in comparison with blood culture [15]. In this sense, Bacconi *et al*. [8] demonstrated that IRIDICA performance was not hampered by the presence of up to 4.0×10^7^ white blood cells/mL. In our study no significant difference in white blood cell count was found between IRIDICA-negative and -positive specimens. However, the two obtained invalid results corresponded to specimens from ICU patients with a high white blood cell count (4.5×10^7^ and 7.2×10^7^ cells/mL). It should also be considered that the IRIDICA software has specific thresholds for reporting different microorganisms and those detections below the threshold are not reported in order to increase the specificity (3–10 genomes/well for most pathogenic bacteria, and 10 genomes/well for those microorganisms that can also be found as skin contaminants). However, this could lead to false negative results in certain cases. In our study, four microorganisms that were considered the etiological agent of the sepsis episode were detected but not reported as they were below those levels (data not shown).

The use of blood culture as gold standard when evaluating molecular methods has limitations, given its low positivity rate (only about 10% of all blood cultures are positive) [19,20]. In this regard, IRIDICA was able to identify an extra 80 microorganisms that did not grow in blood culture. The detection of 41 (51.2%) of them was supported by the clinical condition of the patient or other positive cultures (five microorganisms from four ER patients and 36 microorganisms from 30 ICU patients), and 39 (40%) were easily identified as contaminants (skin or environmental). Finally, there were seven microorganisms (8.8%) that are not commonly found as contaminants and could not be explained by the review of clinical records. These results reinforce the idea that, like the results of the blood culture, the results obtained with molecular methods should be interpreted by the clinician in light of clinical signs and symptoms. Skin or ambient environmental contaminants can also be found in a small percentage of blood cultures due to insufficient aseptic practices during extraction (up to 5% of blood cultures in our setting, which is similar to the 5.4% contamination rate found by IRIDICA). When all the available clinical information was taken into account, IRIDICA showed a positive concordance of 77.2% with respect to clinically diagnosed sepsis. Furthermore, our results are comparable to those recently published by Bacconi *et al*.[8], who described a sensitivity ranging from 83 to 91% in comparison with conventional methods. These results evidence how using a higher volume of whole blood results in an increased detection rate in comparison with the previous version of the technology (50% positive agreement) [6].

The IRIDICA platform has been conceived for the rapid diagnosis of infections in critically ill patients. It should be noticed that this molecular method performed particularly well in the subgroup of ICU patients when compared with the clinical infection criterion gold standard; the sensitivity was 89.0% in the analysis by microorganism *vs*. 73.1% in the ER (*p* = 0.005) and 90.5% in the analysis by specimen *vs*. 73.6% in the ER (*p* = 0.02). The different performance of IRIDICA in the ICU and ER settings could be explained, at least in part, by the inherent characteristics of the patients admitted to the ICU. These patients are severely ill and suffering from underlying pathologies that may increase the risk of developing sepsis. They also have a major risk of suffering from nosocomial infections due to the use of several intravascular devices, such as catheters. Besides, patients staying at the ICU setting for a long period of time may suffer from immunological impairment. All these factors may be related to the presence of higher bacterial loads (a significantly higher number of genomes per well was observed in ICU patients in comparison with those from the ER). Interestingly, the agreement between IRIDICA and the clinical infection criterion was higher than with the blood culture (κ = 0.711 *vs*. κ = 0.315), which points to the presence of clinically relevant microorganisms detected only by the molecular method (73.3% of these patients were under antimicrobial therapy). Finally, IRIDICA showed a negative predictive value of 95% in patients admitted to the ICU, indicating that this technology could be useful for ruling out infection in this setting when the clinical suspicion of sepsis is low.

The ability of detecting a broad range of pathogens was demonstrated in this study, as IRIDICA was able to detect 43 different species of bacteria and *Candida* spp. Interestingly, this technology also detected certain microorganisms that are not commonly recovered from blood culture, such as *Mycoplasma hominis* and *Mycobacterium simiae*. There were six cases reported by the software as bacteria or fungus “detected but not identified” (three of each, respectively). Those detections may indicate the presence of a microorganism that is not usually pathogenic (in the case of bacteria), or that the software does not have enough information to assign an identification (for instance, filamentous fungi are not specifically targeted by the assay but can be amplified by the primers directed to conserved ribosomal genes used for the detection of *Candida* spp.). While most of these cases (three bacteria and one fungus) were considered contaminants, in two cases the detection could have been due to the presence of *Aspergillus fumigatus* in blood, as both patients had respiratory cultures positive for this fungus and also had a positive galactomannan antigen detection. Given that aspergillosis can be a serious complication in immunocompromised patients, obtaining this information (fungus detected but not identified) may be useful in order to run additional confirmatory tests and guide treatment.

This study has several limitations. First, only the specimens from patients admitted to the ICU were consecutively included; while we obtained a dedicated blood specimen from most of the patients admitted to the ER, only those with a paired positive blood culture were included, in order to make the study feasible. Thus, the specimen set did not reflect the usual blood culture positivity rate in the clinical setting (around 10% in our hospital). Nevertheless, the ICU subanalysis does reflect the positivity rate in this department (7.5–13.7%), and no other studies have tested as many patients with a positive blood culture using this technology. Secondly, the specimens were analyzed retrospectively. Thus, we were not able to perform any further testing when discrepancies were found, especially in those involving resistance markers. Although it was surprising that the resistance markers *van*A and *van*B were detected in susceptible *Enterococcus faecium* isolates, this phenomenon has already been described when point mutations or deletions affect the regulatory genes *van*S and *van*R [21,22]. Thirdly, the specimens were stored for varying periods of time at -20°C until tested at IBIS Biosciences. However, long-term stability of whole blood samples under these storage conditions had previously been demonstrated by the manufacturer on spiked samples (unpublished data), and statistical analysis ruled out any significant association between the storage time and the IRIDICA positivity rate. Finally, in some cases blood samples were drawn when the patients were already under antibiotic treatment, which could have led to the detection of clinically relevant microorganisms by the molecular method in patients with a negative BC.

When implementing molecular methods in the clinical microbiology laboratory, cost-effectiveness studies are necessary given that molecular methods are more expensive than conventional ones. However, a rapid identification of the pathogen may lead to the optimization of the administered therapy and, thus, to a prompter recovery of the patient and a shorter stay at the ICU department. Although prospective cost/benefit studies are needed to assess the real impact of this technology in the management of septic patients, significant economic savings have been reported for the molecular SeptiFast assay (Roche, Manheim, Germany) due to the shortening of the ICU stay and a more rational use of antibiotics [23,24].

In conclusion, the IRIDICA technology offers a rapid and reliable identification of pathogens for the diagnosis of sepsis directly from the patient’s blood, with a better performance in ICU patients. When used in combination with conventional methods it could lead to an increase in the number of microbiologically confirmed sepsis cases. More importantly, a significant proportion of septic patients would benefit from an early identification of the pathogen leading to a prompter appropriate antibiotic treatment, which relates to patient survival rates.

## Supporting Information

S1 Table Clinical review of the discrepancies between IRIDICA and blood culture. (DOC)

S2 Table Microorganisms detected by IRIDICA considered as skin or ambient contaminants. (DOCX)

S3 Table Polymicrobial infections by either conventional or molecular methods (*n* = 28). (DOCX)
