# Supplementary material for: Evaluation of the Broad-Range PCR/ESI-MS Technology in Blood Specimens for the Molecular Diagnosis of Bloodstream Infections
Source: PLoS One. 2015 Oct 16;10(10):e0140865. doi: 10.1371/journal.pone.0140865 (PMC4608784; doi:10.1371/journal.pone.0140865)
Supplement: S1 Table — (DOC) [file pone.0140865.s001.doc]

**S1 Table. Clinical review of the discrepancies between IRIDICA and blood culture.**

| **Patient**  **No.** | **Department** | **Blood culture result** | **IRIDICA result** | **QC score/ level*** | **Comments** |
| --- | --- | --- | --- | --- | --- |
| ***Clinical evidence supporting IRIDICA results*** | | | | | |
| **1** | **ER** | *Escherichia coli* | *Escherichia coli* | 0.94/19 | Sepsis of abdominal origin. |
|  |  | *Enterobacter aerogenes* |  |  |  |
|  |  | *Enterococcus casseliflavus* |  |  |  |
|  |  |  | *Klebsiella pneumoniae* | 0.99/174 |  |
| **2** | **ER** | *Escherichia coli* | *Escherichia coli* | 0.92/3 | Sepsis of abdominal origin. |
|  |  |  | *Klebsiella pneumoniae* |  |  |
| **3** | **ER** | *Candida parapsilosis* | *Candida parapsilosis* | 0.99/177 | Patient with a solid neoplasm and sepsis of abdominal origin. |
|  |  | *Enterobacter aerogenes* | *Enterobacter aerogenes* | 0.97/19 |
|  |  |  | *Klebsiella oxytoca* | 0.88/5 |
| **4** | **ER** | *Streptococcus constellatus* |  |  | Patient with peritonitis and sepsis of abdominal origin. Culture of ascetic liquid positive for *E. coli* three days after*.* |
|  |  |  | *Escherichia coli* | 0.96/3 |
|  |  |  | *Fusobacterium nucleatum* | 0.97/37 |
| **5** | **ICU** | Negative | *Candida tropicalis* | 0.96/123 | Previous blood culture positive for *C. tropicalis*. Under antifungal treatment. |
| **6** | **ICU** | Negative | *Enterobacter cloacae complex* | 0.93/3 | Patient with a pneumonia caused by bronchoaspiration. |
|  |  |  | *Streptococcus pneumoniae* | 0.96/12 |
| **7** | **ICU** | Negative | *Enterobacter cloacae complex* | 0.96/86 | Patient with several cultures positive for *E. cloacae* after surgery (bronchial aspirate, urine, wound suppuration). Under antibiotic treatment. |
|  |  |  | *Enterobacter cancerogenus* | 0.94/40 |
| **8** | **ICU** | Negative | *Staphylococcus aureus* | 0.99/27 | Previous blood culture positive for *S. aureus*. Under antibiotic treatment. |
| **9** | **ICU** | Negative | *Haemophilus influenzae* | 0.97/12 | Patient with a pneumonia caused by bronchoaspiration. |
| **10** | **ICU** | Negative | *Mycobacterium simiae* | 0.95/63 | Leukaemia and neutropenia. Acute enterocolitis and severe respiratory insuffiency. |
| **11** | **ICU** | Negative | *Streptococcus pneumoniae* | 0.98/209 | Previous blood culture positive for *S. pneumoniae*. Under antibiotic treatment. |
| **12** | **ICU** | Negative | *Escherichia coli* | 0.96/4 | Intra-abdominal infection. Under antibiotic treatment. |
| **13** | **ICU** | Negative | *Klebsiella pneumoniae* | 0.98/16 | Acute pancreatitis. Abdominal fluid culture positive for *K. pneumoniae.* Under antibiotic treatment. |
| **14** | **ICU** | Negative | *Escherichia coli* | 0.99/388 | Patient with acute myeloid limfoma. Previous cultures positive for *E. coli* (pleural liquid and blood culture). Under antibiotic treatment. |
| **15** | **ICU** | Negative | *Pseudomonas aeruginosa* | 0.99/38 | Previous respiratory specimen positive for *P. aeruginosa.* Under antibiotic treatment. |
| **16** | **ICU** | Negative | *Staphylococcus aureus* | 0.97/8 | Endocarditis by *S. aureus.* Under antibiotic treatment. |
| **17** | **ICU** | Negative | *Pseudomonas aeruginosa* | 0.98/190 | Sepsis of respiratory origin. Mechanical ventilation. Under antibiotic treatment. |
| **18** | **ICU** | Negative | *Escherichia coli* | 0.96/68 | Nephrolithiasis with a urethral obstruction. |
|  |  |  | *Escherichia coli/Shigella spp.* | 0.95/79 |
| **19** | **ICU** | Negative | *Mycobacterium simiae* | 0.86/11 | Alcoholic cirrhosis. Under antibiotic treatment. |
| **20** | **ICU** | Negative | *Escherichia coli/Shigella spp.* | 0.96/26 | Patient with a pneumonia caused by bronchoaspiration. Under antibiotic treatment. |
| **21** | **ICU** | Negative | *Escherichia coli* | 0.97/4 | Sepsis of abdominal origin. Purulent peritonitis. Under antibiotic treatment. |
| **22** | **ICU** | Negative | *Enterococcus faecium* | 0.98/42 | Patient with acute myeloid lymphoma. Neutropenic enterocolitis. Respiratory specimen positive for *C. albicans* 6 days after the specimen tested by IRIDICA. Under antibiotic (no antifungal) treatment. |
|  |  |  | *Candida albicans* | 0.99/57 |
| **23** | **ICU** | Negative | *Streptococcus dysagalactiae* | 0.99/157 | Patient with multiple myeloma and aplasia. Sepsis of respiratory origin. Previous blood culture positive for *S. dysagalactiae.* Respiratory specimen positive for *Aspergilllus* spp. and galactomannan antigen positive a few days later. Under antibiotic treatment. |
|  |  |  | *Fungus detected. No ID provided* | 0.95/15 |
| **24** | **ICU** | Negative | *Fusobacterium nucleatum* | 0.98/24 | Patient with acute myeloid lymphoma and neutropenia presenting severe mucositis. Under antibiotic treatment. |
| **25** | **ICU** | Negative | *Fusobacterium nucleatum* | 0.97/9 | Patient presenting acute peritonitis. |
| **26** | **ICU** | Negative | *Prevotella denticola* | 0.94/13 | Sepsis of abdominal origin. Purulent peritonitis. Under antibiotic treatment. |
| **27** | **ICU** | Negative | *Streptococcus oralis/pneumoniae* | 0.97/24 | Meningitis by *S. pneumoniae.* Under antibiotic treatment. |
| **28** | **ICU** | Negative | *Pseudomonas aeruginosa* | 0.99/480 | Sepsis of abdominal origin. |
| **29** | **ICU** | Negative | *Streptococcus pyogenes* | 0.96/29 | Sepsis due to cellulitis. Under antibiotic treatment. |
| **30** | **ICU** | *Elisabethkingia meningoseptica* | *Elisabethkingia meningoseptica* | 0.97/39 | Patient with neutropenia. Sepsis of primary origin. Under antibiotic (no antifungal) treatment. |
|  |  |  | *Candida albicans* | 0.97/3 |
|  |  | Negative | *Mycoplasma hominis* | 0.99/105 | Vulvar abcess (second specimen from the same episode). Under antibiotic treatment. |
| **31** | **ICU** | *Streptococcus mitis* | *Streptococcus mitis* | 0.97/152 | Blood culture positive for *S. mitis.* |
|  |  |  | *Streptoccus spp.* | 0.97/31 |
| **32** | **ICU** | *Enterococcus faecium* | *Enterococcus faecium* | 0.97/58 | Sepsis of primary origin. Respiratory specimen positive for *Aspergilllus* spp. and galactomannan antigen positive. Under antibiotic treatment. |
|  |  |  | *Fungus detected. No ID provided* | 0.97/58 |
| **33** | **ICU** | *Candida albicans* |  |  | Respiratory culture positive by methicillin-resistant *S. aureus* (MRSA). Under antibiotic treatment. |
|  |  |  | *Staphylococcus aureus mecA* | 0.96/7 |
| **34** | **ICU** | *Pseudomonas aeruginosa* | *Pseudomonas aeruginosa* |  | Sepsis of abdominal origin. |
|  |  |  | *Klebsiella oxytoca* | 0.98/81 |
| ***No clinical evidence supporting IRIDICA results*** | | | | | |
| **1** | **ICU** | Negative | *Escherichia coli* | 0.97/17 | Patient with a cranioencephalic traumatism. Respiratory culture positive by *S. constellatus.* Under antibiotic treatment. |
| **2** | **ICU** | *Staphylococcus aureus* |  |  | Sepsis due to *S. aureus.* |
|  |  |  | *Fusobacterium nucleatum* | 0.96/80 |
| **3** | **ER** | Negative | Methicillin-resistant *S. aureus* (MRSA) | 0.91/1 | Patient with multiple myeloma and aplasia. Sepsis of respiratory origin (same specimen than case 23) |
| **4** | **ER** | *Escherichia coli* | *Escherichia coli* | 0.97/15 | Sepsis of urologic origin. |
|  |  |  | *Enterobacter cloacae complex* | 0.90/7 |
| **5** | **ER** | *Enterococcus gallinarum* |  |  | Sepsis of urologic origin. |
|  |  |  | *Clostridium perfingens* | 0.97/53 |
| **6** | **ER** | Negative | *Citrobacter freundii* |  | Sepsis of urologic origin. |
| **7** | **ER** | *Escherichia coli* | *Escherichia coli* | 0.96/42 | Sepsis of urologic origin. Possible misidentification due to high genetic similarity. |
|  |  |  | *Shigella boydii* | 0.95/5 |

ER, Emergency Room; ICU, Intensive Care Unit; QC score, quality score (relative measure of the strength of the data supporting identification); Level, number of genomes/well.
