# Supplementary material for: Evaluation of the Broad-Range PCR/ESI-MS Technology in Blood Specimens for the Molecular Diagnosis of Bloodstream Infections
Source: PLoS One. 2015 Oct 16;10(10):e0140865. doi: 10.1371/journal.pone.0140865 (PMC4608784; doi:10.1371/journal.pone.0140865)
Supplement: S2 Table — (DOCX) [file pone.0140865.s002.docx]

**S2 Table. Microorganisms detected by IRIDICA considered as skin or ambient contaminants.**

| **Microorganism** | **N** |
| --- | --- |
| Coagulase-negative staphylococci | 4 |
| *Micrococcus luteus* | 2 |
| *Corynebacterium* spp. | 2 |
| *Streptococcus* spp. | 1 |
| *Propionibacterium acnes* | 10 |
| *Acinetobacter johnsonii* | 1 |
| *Acinetobacter junii* | 1 |
| *Pseudomonas entomophila/putida* | 1 |
| *Pseudomonas mendocina* | 1 |
| *Pectobacterium carotovum* | 1 |
| *Sphinobacterium multivorum* | 1 |
| *Methylobacterium zatmanii* | 1 |
| Bacteria detected. No ID provided | 3 |
| Fungus detected. No ID provided | 3 |
| **TOTAL** | **32** |
