# Supplementary material for: Evaluation of the Broad-Range PCR/ESI-MS Technology in Blood Specimens for the Molecular Diagnosis of Bloodstream Infections
Source: PLoS One. 2015 Oct 16;10(10):e0140865. doi: 10.1371/journal.pone.0140865 (PMC4608784; doi:10.1371/journal.pone.0140865)
Supplement: S3 Table — (DOCX) [file pone.0140865.s003.docx]

**S3 Table. Polymicrobial infections by either conventional or molecular methods (*n*=28).**

|  | **Department** | **Conventional methods** | **IRIDICA** |
| --- | --- | --- | --- |
| **Concordant ID by blood culture and IRIDICA** | | | |
| 1 | **ER** | *Proteus mirabilis* | *Proteus mirabilis* |
|  |  | *Enterococcus faecalis* | *Enterococcus faecalis* |
| 2 | **ER** | *Pseudomonas aeruginosa* | *Pseudomonas aeruginosa* |
|  |  | *Streptococcus viridans* | *Streptococus* spp. |
| 3 | **ER** | *Escherichia coli* | *Escherichia coli* |
|  |  | Not isolated | *Escherichia coli/Shigella* spp. |
| 4 | **ER** | *Enterobacter cloacae* | *Enterobacter cloacae* complex |
|  |  | *Enterococcus faecium* | *Enterococcus faecium* |
| 5 | **ER** | *Escherichia coli* | *Escherichia coli* |
|  |  | Not isolated | *Escherichia coli/Shigella* spp. |
| 6 | **ICU** | *Stenotrophomonas maltophilia* | *Stenotrophomonas maltophilia* |
|  |  | *Candida tropicalis* | *Candida tropicalis* |
| 7 | **ICU** | *Streptococcus mitis* | Viridans/mitis group |
|  |  | Not isolated | *Streptoccus* spp. |
| 8 | **ICU** | *Enterococcus faecium* | *Enterococcus faecium* |
|  |  | Coagulase-negative Staphylococci | *Staphylococcus epidermidis* |
|  |  |  | *Staphylococcus haemolyticus* |
| **Blood culture detected more microorganisms than IRIDICA** | | | |
| 1 | **ER** | *Escherichia coli* | *Escherichia coli* |
|  |  | *Proteus mirabilis* | Not detected |
| 2 | **ER** | *Escherichia coli* | *Escherichia coli* |
|  |  | *Enterococcus faecalis* | Not detected |
|  |  | *Enterobacter cloacae* | Not detected |
| 3 | **ER** | *Klebsiella pneumoniae* | *Klebsiella pneumoniae* |
|  |  | *Escherichia coli* | Escherichia coli |
|  |  | *Enterococcus gallinarum* | Not detected |
| 4 | **ER** | *Klebsiella pneumoniae* | *Klebsiella pneumoniae* |
|  |  | *Enterococcus gallinarum* | Not detected |
| 5 | **ER** | *Escherichia coli* | *Escherichia coli* |
|  |  | *Enterobacter aerogenes* | Not detected |
|  |  | *Enterococcus casselifavus* | Not detected |
|  |  | Not isolated | *Klebsiella pneumoniae* |
| **IRIDICA detected more microorganisms than blood culture** | | | |
| 1 | **ER** | *Candida parapsilosis* | *Candida parapsilosis* |
|  |  | *Enterobacter aerogenes* | *Enterobacter aerogenes* |
|  |  | Not isolated | *Klebsiella oxytoca* |
| 2 | **ER** | *Escherichia coli* | *Escherichia coli* |
|  |  | Not isolated | *Klebsiella pneumoniae* |
| 3 | **ICU** | *Enterococcus faecium* | *Enterococcus faecium* |
|  |  | Not isolated | Fungus detected. No ID provided |
| 4 | **ICU** | *Pseudomonas aeruginosa* | *Pseudomonas aeruginosa* |
|  |  | Not isolated | *Klebsiella oxytoca* |
| 5 | **ICU** | *Elisabethkingia meningoseptica* | *Elisabethkingia meningoseptica* |
|  |  | Not isolated | *Candida albicans* |
| **Blood culture and IRIDICA detected different microorganisms** | | | |
| 1 | **ER** | *Streptococcus constellatus* | Not detected |
|  |  | Not isolated | *Escherichia coli* |
|  |  | Not isolated | *Fusobacterium nucleatum* |
| 2 | **ICU** | *Candida albicans* | Not detected |
|  |  | Not isolated | *Staphylococcus aureus mec*A |
| 3 | **ICU** | *Candida parapsilosis* | Not detected |
|  |  | Not isolated | *Staphylococcus aureus* |
| **Only by blood culture** | | | |
| 1 | **ER** | *Escherichia coli* | Not detected |
|  |  | *Streptococcus mitis* | Not detected |
| 2 | **ICU** | *Lactobacillus* spp. | Not detected |
|  |  | *Candida glabrata* | Not detected |
| **Only by IRIDICA** | | | |
| 1 | **ICU** | Not isolated | *Enterobacter cloacae complex* |
|  |  | Not isolated | *Enterbacter cancerogenus* |
| 2 | **ICU** | Not isolated | *Escherichia coli* |
|  |  | Not isolated | Escherichia coli/Shigella spp. |
| 3 | **ICU** | Not isolated | *Enterococcus faecium* |
|  |  | Not isolated | *Candida albicans* |
| 4 | **ICU** | Not isolated | *Streptococcus dysagalactiae* |
|  |  | Not isolated | Fungus detected. No ID provided |
| 5 | **ICU** | Not isolated | *Enterobacter cloacae complex* |
|  |  | Not isolated | *Streptococcus pneumoniae* |

ER, Emergency Room; ICU, Intensive Care Unit.
